# Generic utilities in chronic obstructive pulmonary disease patients stratified according to different staging systems

**DOI:** 10.1186/s12955-014-0120-5

**Published:** 2014-09-05

**Authors:** Marc Miravitlles, Alicia Huerta, José Alberto Fernández-Villar, Bernardino Alcázar, Guillermo Villa, Carles Forné, Maribel Cuesta, Carlos Crespo, Francisco García-Río

**Affiliations:** Pneumology Department, Hospital Universitari Vall d’Hebron. CIBER de Enfermedades Respiratorias (CIBERES), Pg. Vall d’Hebron 119-129, Barcelona, 08035 Spain; Market Access Department, GlaxoSmithKline, C/Severo Ochoa 2, Tres Cantos, Madrid, 28760 Spain; Pneumology Department, Complexo Hospitalario Universitario de Vigo, Calle Rúa Pizarro 22, Pontevedra Vigo, 36036 Spain; Pneumology Department, Hospital de Alta Resolución de Loja, Avda. Tierno Galván s/n, Loja Granada, 18300 Spain; Oblikue Consulting, C/Josep Irla i Bosch, 5-7, Barcelona, 08034 Spain; Department of Statistics, University of Barcelona, C/Diagonal 643, Barcelona, 08028 Spain; Pneumology Department, Hospital Universitario La Paz, IdiPAZ, Paseo de la Castellana 261, Madrid, 28046 Spain

**Keywords:** Chronic obstructive pulmonary disease, Health utility, Health related quality of life, GOLD, GesEPOC

## Abstract

**Background:**

To determine generic utilities for Spanish chronic obstructive pulmonary disease (COPD) patients stratified by different classifications: GOLD 2007, GOLD 2013, GesEPOC 2012 and BODEx index.

**Methods:**

Multicentre, observational, cross-sectional study. Patients were aged ≥40 years, with spirometrically confirmed COPD. Utility values were derived from EQ-5D-3 L. Means, standard deviations (SD), medians and interquartile ranges (IQR) were computed based on the different classifications. Differences in median utilities between groups were assessed by non-parametric tests.

**Results:**

346 patients were included, of which 85.5% were male with a mean age of 67.9 (SD = 9.7) years and a mean duration of COPD of 7.6 (SD = 5.8) years; 80.3% were ex-smokers and the mean smoking history was 54.2 (SD = 33.2) pack-years. Median utilities (IQR) by GOLD 2007 were 0.87 (0.22) for moderate; 0.80 (0.26) for severe and 0.67 (0.42) for very-severe patients (p < 0.001 for all comparisons). Median utilities by GOLD 2013 were group A: 1.0 (0.09); group B: 0.87 (0.13); group C: 1.0 (0.16); group D: 0.74 (0.29); comparisons were statistically significant (p < 0.001) except A vs C. Median utilities by GesEPOC phenotypes were 0.84 (0.33) for non exacerbator; 0.80 (0.26) for COPD-asthma overlap; 0.71 (0.62) for exacerbator with emphysema; 0.72 (0.57) for exacerbator with chronic bronchitis (p < 0.001). Comparisons between patients with or without exacerbations and between patients with COPD-asthma overlap and exacerbator with chronic bronchitis were statistically-significant (p < 0.001). Median utilities by BODEx index were: group 0–2: 0.89 (0.20); group 3–4: 0.80 (0.27); group 5–6: 0.67 (0.29); group 7–9: 0.41 (0.31). All comparisons were significant (p < 0.001) except between groups 3–4 and 5–6.

**Conclusion:**

Irrespective of the classification used utilities were associated to disease severity. Some clinical phenotypes were associated with worse utilities, probably related to a higher frequency of exacerbations. GOLD 2007 guidelines and BODEx index better discriminated patients with a worse health status than GOLD 2013 guidelines, while GOLD 2013 guidelines were better able to identify a smaller group of patients with the best health*.*

## Background

Chronic obstructive pulmonary disease (COPD) is a highly-prevalent, progressive respiratory disease and a major cause of morbidity and mortality worldwide [1]. Up to 10.2% of adults aged 40–80 years are affected by COPD in Spain [2] and individuals with COPD experience significant impairments in health-related quality of life (HRQL) [3].

There is interest in classifying the severity of COPD in order to tailor diagnostic and therapeutic interventions. In 2007, the Global Initiative for Chronic Obstructive Lung Disease (GOLD) [4] proposed a patient stratification based on the severity of airflow limitation, as measured by forced expiratory volume in 1 second, percentage of predicted (FEV_1_(%)). However, airflow obstruction is only one aspect of COPD, as there are other aspects like reduced muscle strength, dyspnoea or functional impairment in daily activities that are key components of the health status of COPD patients. For this reason, GOLD 2013 update [5] proposed a combined assessment of COPD severity including lung function, the history of exacerbations and the presence of symptoms. Likewise, in 2012, in recognition of the heterogeneity of COPD, the Spanish Society of Pulmonology and Thoracic Surgery, together with the scientific societies involved in the health care of COPD patients, produced new clinical practice guidelines (GesEPOC) [6] based on the clinical phenotypes of COPD and proposed a COPD severity classification based on the BODEx index.

Health utility is a measurement of preference that shows the value placed on different health states over a specific period. It is generally measured on a scale of 0–1, with 0 being the worst possible (death) and 1 the best possible health status [7]. Health utilities are widely used in health economics as they provide information for clinicians, managers and other decision makers on the preferences given to certain health states. Health utilities also allow measuring the benefits of health interventions in terms of quality-adjusted life years, and they constitute an essential parameter in cost-utility (CU) analyses, which are the recommended type of economic evaluation [8,9].

In daily clinical practice, the most common instruments for estimating health utilities are preference-based generic HRQL questionnaires, of which the EuroQol-5D (EQ-5D) [10] is the most-widely used. Furthermore, EQ-5D questionnaire is recommended by health technology assessment bodies like the National Institute for Health and Care Excellence.

Various international [11–14] and Spanish [2,15–18] studies have estimated the utility of COPD patients. However, until now, no study had compared utilities in the same population of COPD patients classified according to different staging systems.

The aim of this study was to determine, using EQ-5D 3 levels (EQ-5D-3 L) questionnaire, health utilities in Spanish COPD patients stratified according to different severity classifications. Primary analysis was performed according to GOLD 2007. Secondary analysis included GOLD 2013, GesEPOC 2012 (phenotypes and BODEx) classifications.

## Methods

Multicentre, observational, cross-sectional study including patients recruited from 15 Spanish hospitals. The study was approved by the Clinical Research and Ethics Committee of Hospital Clinic (Barcelona, Spain) and all patients gave written informed consent to participate.

### Study population

Patients of both sexes, aged ≥40 years, with a diagnosis of COPD of >12 months confirmed by spirometry (post bronchodilator FEV_1_/FVC < 0.70 and FEV_1_ < 80%) were included. All patients who attended to a scheduled outpatient visit and fulfilled the inclusion/exclusion criteria were recruited consecutively by the investigator of each centre until the number of patients necessary for each stratum had been completed. Patients had to be on stable state without any exacerbation or hospitalization in the previous 2 months. Patients with other respiratory disease, advanced cancer (without possibility of remission), terminal patients or receiving palliative care, and patients with cognitive impairment unable to understand or complete the informed consent form and questionnaires, were excluded.

Sample size was estimated for the primary objective of the study and was powered to detect differences in health utilities in COPD patients stratified by lung function impairment: moderate, severe, and very-severe (GOLD 2007) [4]. In order to detect a minimum clinically relevant difference in means of 0.1 points in health utilities [19,20] between two groups of patients (moderate vs. very-severe and severe vs. very-severe) and assuming a standard deviation (SD) of 0.26 points [11], at least 128 patients per group would be required (95% reliability, 90% power, one-sided test, 10% dropout rate). Due to the lower prevalence of very-severe patients, which made recruitment difficult, sample size was recalculated to obtain the minimum number of very-severe patients needed to achieve a statistical power of 80%. After this new calculation, 68 very-severe patients were required, assuming a sample size of 128 patients with moderate and 128 with severe COPD (95% reliability, one-sided test, 10% dropout rate).

### Data collection and measurements

The main demographic and clinical variables, including the clinical phenotype, were collected using a case report form (CRF) specifically designed for the study.

Pre-and post-bronchodilator lung function data (FEV_1_) were obtained from spirometric testing (last measurement performed in the previous 12 months or, if not available, performed during the inclusion visit).

Comorbidities were evaluated by the Charlson comorbidity index [21], which predicts 10-year mortality for a patient who may have a range of comorbid conditions. Each condition is assigned a score (1, 2, 3 or 6) depending on the associated risk of death.

In addition, each patient completed the EQ-5D-3 L questionnaire as well as the COPD Assessment Test (CAT) [22] and the modified Medical Research Council Dyspnoea Scale (mMRC) [23], both required for GOLD 2013 classification.

EQ-5D-3 L is a preference-based generic HRQL questionnaire consisting of 5 dimensions relating to health (mobility, self-care, usual activities, pain/discomfort and anxiety/depression) and ranges between 5 and 15. Each dimension is divided into three levels of functioning (no problems, some problems and extreme problems). Respondents are asked to describe their health status by ticking off one level of functioning for each of the five dimensions, generating up to 243 different health states. The questionnaire also includes a Visual Analogue Scale (VAS) in which respondents are asked to value their overall health status on a scale from 0 (worst imaginable health state) to 100 (best imaginable health state).

CAT is a COPD-specific questionnaire that measures the impact of the disease on HRQL and allows symptoms to be described. It evaluates the following symptoms: ongoing cough, breathlessness, wheezing, chest tightness, impairment in daily activities, confidence, quality of sleep and energy. CAT score ranges from 0 to 40, with 0 representing the lowest impact on HRQL and 40 the maximum impact.

mMRC measures the impact of dyspnoea on the activities of daily living. The score ranges from 0 (no dyspnoea) to 4 (dyspnoea preventing the patient leaving home or which appears with activities such as dressing or undressing).

Patients were stratified according to the phenotype or severity criteria listed in clinical guidelines:

- GOLD 2007 [4]: Severity was determined according to the post-bronchodilator FEV_1_ using the following criteria: moderate, 50% ≤ FEV_1_ < 80% predicted; severe, 30% ≤ FEV_1_ < 50% predicted; and very-severe, FEV_1_ < 30% predicted.
- GOLD 2013 [5]: COPD patients were categorized in four groups depending on the level of risk and symptoms: A (low risk, fewer symptoms), B (low risk, more symptoms), C (high risk, fewer symptoms) D (high risk, more symptoms). Risk was assessed according to the spirometric classification (high risk: severe and very-severe) and/or total exacerbations in the past year (high risk: ≥ 2 exacerbations). In addition, patients with ≥1 hospitalizations for COPD exacerbations were considered high risk. Symptoms were assessed according to mMRC and CAT. In case of discrepancy between mMRC and CAT results, the highest level of severity was chosen. However, a sensitivity analysis was performed by classifying patients using only CAT or using only mMRC.
- Clinical phenotypes [6]: Phenotype characterization was collected in the corresponding CRFs according to GesEPOC criteria: non exacerbator phenotype, corresponding to patients with less than 2 exacerbations the previous year; COPD-asthma overlap phenotype or patients with a previous diagnosis of asthma or some characteristics of asthma, such as very positive bronchodilator test, high serum IgE levels, increased blood eosinophilia and others according to a consensus criteria recently published [24]; exacerbator with emphysema phenotype or patients with 2 or more exacerbations the previous year and diagnosed with clinical/radiological/functional emphysema; and exacerbator with chronic bronchitis phenotype or patients with 2 or more exacerbations the previous year and chronic cough and sputum production.
- BODEx index [25]: This multidimensional scale categorizes patients in four levels from lesser to greater severity (0–2, 3–4, 5–6 and 7–9) according to BMI, FEV_1_ (%) post-bronchodilator, severe exacerbations and mMRC score values.

### Statistical analysis

Descriptive statistics included means, SD, medians and interquartile ranges (IQR) for continuous variables and absolute frequencies and percentages for discrete variables.

Health utilities were derived from EQ-5D-3 L scores by applying weighted Spanish societal preferences [26] to EQ-5D patient scores according to the following formula:

$$ \begin{array}{l}\mathrm{Utility} = 1\hbox{-} 0.024\hbox{-} 0.106\cdotp \left(\mathrm{Mobility} = 2\right)\hbox{-} 0.430 \cdot p\ \left(\mathrm{Mobility} = 3\right)\hbox{-} 0.134 \cdot p\ \\ {}\left(\mathrm{Self}\hbox{-} \mathrm{care} = 2\right)\hbox{-} 0.309\cdotp \left(\mathrm{Self}\hbox{-} \mathrm{care} = 3\right)\hbox{-} 0.071\cdotp \left(\mathrm{Activity} = 2\right)\hbox{-} 0.195 \cdot p\ \left(\mathrm{Activity} = 3\right)\\ {}\hbox{-} 0.089\cdotp \left(\mathrm{Pain} = 2\right)\hbox{-} 0.261 \cdot p\ \left(\mathrm{Pain} = 3\right)\hbox{-} 0.062 \cdot p\ \left(\mathrm{Anxiety} = 2\right)\hbox{-} 0.144 \cdot p\ \left(\mathrm{Anxiety} = 3\right)\\ {}\hbox{-} 0.291\cdotp \left(\mathrm{if}\ \mathrm{at}\ \mathrm{least}\ \mathrm{one}\ \mathrm{answer}\ \mathrm{is}\ 3\right) + 0.024 \cdot p\ \left(\mathrm{if}\ \mathrm{all}\ \mathrm{answer}\mathrm{s}\ \mathrm{are}\ 1\right).\end{array} $$

Descriptive analysis of health utilities were calculated for the different groups defined. Differences between groups were assessed using non-parametric tests (Kruskal-Wallis, Mann–Whitney U) due to the negative asymmetry shown by EQ-5D utility values (*ceiling effect*) [12,13,20,27]. Post-hoc comparisons were performed using the Bonferroni correction for multiple testing. Statistical significance was established as alpha = 0.05 for all analyses. R version 3.0.2 statistical package [28] was used.

## Results

A total of 358 patients were recruited, of whom 346 met all the selection criteria and were included in the final analysis.

Patients were 85.5% male, with a mean age of 67.9 (SD = 9.7) years and a mean duration of COPD of 7.6 (SD = 5.8) years; 80.3% were ex-smokers and the mean smoking history was 54.2 (SD = 33.2) pack-years. Mean post bronchodilator FEV_1_ (%) was 46.2% (SD = 12.1%). The most frequent comorbidities were cardiovascular diseases (27.2%) and diabetes (16.5%). The mean Charlson index was 1 (SD = 1.4). In the previous 12 months, 58.4% of patients had suffered at least one exacerbation, with a mean of 1.3 (SD = 1.5) exacerbations. Mean scores for CAT, mMRC and BODEx index were 16.2 (SD = 7.8), 1.8 (SD = 1.1), and 2.9 (SD = 1.9), respectively (Table 1). Table 2 shows the cross-tabulated distribution of patients according to the different classifications used. GOLD 2013 guidelines could differentiate patients with moderate COPD, while the BODEx index differentiated better patients with severe or very-severe COPD. GesEPOC phenotypes differentiated between COPD patients regardless of their GOLD 2007 classification.

**Table 1:** **Characteristics of the study population**

**Characteristic**	**Statistics**	**N**
Age (years), mean (SD)	67.9 (9.7)	346
Male, n (%)	296 (85.5%)	346
Smoker, n (%)	68 (19.7%)	346
Former smoker, n (%)	278 (80.3%)	
Pack-years^1^, mean (SD)	54.2 (33.2)	346
Time from diagnosis (years), mean (SD)	7.6 (5.8)	346
BMI (kg/m^2^), mean (SD)	28.0 (5.4)	346
Lung function (post-bronchodilator), mean (SD)		346
*FVC (ml)*	2619.3 (781.8)	
*FVC (%)*	72.5 (18.7)	
*FEV*_*1*_ *(ml)*	1272.1 (506.0)	
*FEV*_*1*_ *(%)*	46.2 (15.5)	
*FEV*_*1*_ */FVC (%)*	48.5 (11.6)	
Positive post-bronchodilator test, n (%)	41 (15.9%)	258
Comorbidities (the most prevalent), n (%)		346
Cardiovascular disease^2^	94 (27.17%)	
Diabetes^2^	57 (16.47%)	
Malignant neoplasias	43 (12.43%)	
Charlson index, mean (SD)	1.0 (1.4)	346
At least one exacerbation previous year, n (%)	202 (58.4%)	346
Exacerbations in the previous year, mean (SD)	1.3 (1.5)	346
At least one admission in previous year, n (%)	62 (17.9%)	346
Admissions previous year, mean (SD)	0.3 (0.6)	346
CAT total score^3^, mean (SD)	16.2 (7.8)	346
Impact in CAT, n (%)		346
Mild (0–10)	74 (21.4%)	
Moderate (10–20)	163 (47.1%)	
Severe (20–30)	87 (25.1%)	
Very-severe (30–40)	22 (6.4%)	
mMRC total score^4^	1.8 (1.1)	346
Impact in mMRC, n (%)		346
0	26 (7.5%)	
1	129 (37.3%)	
2	95 (27.5%)	
3	69 (19.9%)	
4	27 (7.8%)	
BODEx index, mean (SD)	2.9 (1.9)	346
BODEx index classification, n (%)		346
0-2	167 (48.3%)	
3-4	93 (26.9%)	
5-6	75 (21.7%)	
7-9	11 (3.2%)	

^1^Number of pack years = (packs smoked per day) × (years as a smoker).

^2^Cardiovascular diseases are myocardial infarction, congestive heart failure, peripheral vascular disease and cerebrovascular disease. Diabetes means mild or moderate diabetes and diabetes with chronic complications.

^3^Range of CAT total score is 0–40.

^4^Range of mMRC total score is 0–4.

SD = standard deviation, IQR = interquartile range, BMI = body mass index.

**Table 2:** **GOLD 2013, clinical phenotypes and BODEx index by GOLD 2007 classification**

		**GOLD 2007** ^**1**^		
		**Moderate n (%)**	**Severe n (%)**	**Very-severe n (%)**
**GOLD 2013** ^**2**^	**A**	28 (20.7%)	0 (0.0%)	0 (0.0%)
	**B**	66 (48.9%)	0 (0.0%)	0 (0.0%)
	**C**	6 (4.4%)	22 (15.2%)	2 (3.0%)
	**D**	35 (25.9%)	123 (84.8%)	64 (97.0%)
**Clinical phenotype (GesEPOC)**	**Non exacerbator**	86 (68.8%)	88 (64.2%)	33 (55.0%)
	**COPD-asthma overlap**	13 (10.4%)	7 (5.1%)	1 (1.7%)
	**Exacerbator with emphysema**	9 (7.2%)	18 (13.1%)	14 (23.3%)
	**Exacerbator with chronic bronchitis**	17 (13.6%)	24 (17.5%)	12 (20.0%)
**BODEx index classification**	**0-2**	124 (91.9%)	43 (29.7%)	0 (0.0%)
	**3-4**	9 (6.7%)	72 (49.7%)	12 (18.2%)
	**5-6**	2 (1.5%)	27 (18.6%)	46 (69.7%)
	**7-9**	0 (0.0%)	3 (2.1%)	8 (12.1%)

^1^COPD moderate: FEV1/FVC < 0.70 and 50% ≤ FEV1 < 80%*, COPD severe: FEV1/FVC < 0.70 and 30% ≤ FEV1 < 50%*, COPD very-severe: FEV1/FVC < 0.70 y FEV1 < 30%*. *respect to the reference value.

^2^A: low risk, few symptoms; B: low risk, many symptoms; C: high risk, few symptoms; D: high risk, many symptoms.

### Determination of health utilities

According to EQ-5D-3 L results, the most frequent profile was a COPD patient with some problems in walking (53.2%), no problems in self-care (71.1%), no problems in performing usual activities (58.1%), without pain or discomfort (69.7%), and without symptoms of anxiety or depression (57.5%). EQ-5D mean total score was 7.2 (SD = 2.0), and the mean VAS score was 59.1 (SD = 18.5) (Table 3).

**Table 3:** **EQ-5D-3 L quality of life questionnaire and utility values**

**EQ-5D-3 L**	**n (%)**		**N**
**Mobility**			346
***No problems***	160 (46.2%)		
***Some problems***	184 (53.2%)		
***Extreme problems***	2 (0.6%)		
**Self-care**			
***No problems***	246 (71.1%)		
***Some problems***	83 (24.0%)		
***Extreme problems***	17 (4.9%)		
**Usual activities**			
***No problems***	201 (58.1%)		
***Some problems***	123 (35.5%)		
***Extreme problems***	22 (6.4%)		
**Pain/discomfort**			
***No problems***	241 (69.7%)		
***Some problems***	97 (28.0%)		
***Extreme problems***	8 (2.3%)		
**Anxiety/depression**			
***No problems***	199 (57.5%)		
***Some problems***	115 (33.2%)		
***Extreme problems***	32 (9.2%)		
	**Mean (SD)**	**Median (IQR)**	**N**
**Overall health status (range 1–15)**	7.2 (2.0)	7.0 (2.0)	346
**VAS (range 1–100)**	59.1 (18.5)	60.0 (20.0)	345
**Utility values**	0.73 (0.29)	0.81 (0.26)	346

SD = standard deviation, IQR = interquartile range, VAS = visual analogic scale.

The mean value of EQ-5D health utilities was 0.73 (SD = 0.29), and the median was 0.81 (IQR = 0.26). The health utilities obtained showed substantial negative asymmetry (ceiling effect). The percentage of patients with the highest-possible utility was 22%. This percentage decreased with increasing disease severity (29.6% in patients with moderate COPD, 20% in patients with severe COPD and 10.6% in patients with very-severe COPD).

### Utilities according to GOLD 2007

**Figure 1:**
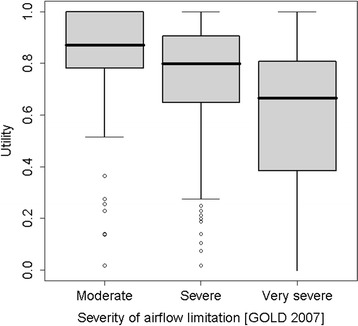
**Utility estimates by GOLD 2007 classification.** LEGEND: Graph shows utilities ≥ 0.

135 (39.0%) moderate, 145 (41.9%) severe and 66 (19.1%) very-severe patients were included. Stratified analysis of health utilities according to pulmonary function showed significant differences between the three groups (Table 4 and Figure 1). There was a significant trend to lower health utilities as pulmonary function declined.

**Table 4:** **Utility estimates by GOLD 2007, GOLD 2013, clinical phenotypes and BODEx index classification**

		**Utilities**			**p** ^**1**^
		**n (%)**	**Mean (SD)**	**Median (IQR)**	
**GOLD 2007** ^**2**^	***COPD moderate***	135 (39.0%)	0.82 (0.22)	0.87 (0.22)	<0.001
	***COPD severe***	145 (41.9%)	0.72 (0.29)	0.80 (0.26)	
	***COPD very-severe***	66 (19.1%)	0.57 (0.35)	0.66 (0.42)	
**GOLD 2013** ^**3**^	***A***	28 (8.1%)	0.96 (0.07)	1.00 (0.09)	<0.001
	***B***	66 (19.1%)	0.80 (0.22)	0.87 (0.13)	
	***C***	30 (8.7%)	0.88 (0.25)	1.00 (0.16)	
	***D***	222 (64.2%)	0.66 (0.31)	0.74 (0.29)	
**Clinical phenotype (GesEPOC)**	***Non exacerbator***	207 (64.3%)	0.79 (0.24)	0.84 (0.33)	<0.001
	***COPD-asthma overlap***	21 (6.5%)	0.81 (0.20)	0.80 (0.26)	
	***Exacerbator with emphysema***	41 (12.7%)	0.56 (0.42)	0.71 (0.62)	
	***Exacerbator with chronic bronchitis***	53 (16.5%)	0.59 (0.36)	0.72 (0.57)	
**BODEx index classification**	***0-2***	167 (48.3%)	0.85 (0.18)	0.89 (0.20)	<0.001
	***3-4***	93 (26.9%)	0.69 (0.29)	0.80 (0.27)	
	***5-6***	75 (21.7%)	0.56 (0.35)	0.67 (0.29)	
	***7-9***	11 (3.2%)	0.33 (0.30)	0.41 (0.31)	

^1^Kruskal-Wallis test and Mann–Whitney *U* test for post-hoc comparisons (Bonferroni-adjusted). Unilateral tests for pulmonary function, GesEPOC and BODEx index.

GOLD 2007: all pairwise comparisons were significant; GOLD 2013: all pairwise comparisons were significant, except for the comparison between groups A and C; Clinical phenotypes: differences between phenotypes A and C, A and D, and between B and D; BODEx index classification: all pairwise comparisons were significant except between the two groups with the greatest severity.

^2^COPD moderate: FEV_1_/FVC < 0.70 and 50% ≤ FEV_1_ < 80%*, COPD severe: FEV_1_/FVC < 0.70 and 30% ≤ FEV_1_ < 50%*, COPD very-severe: FEV_1_/FVC < 0.70 y FEV_1_ < 30%*. *respect to the reference value.

^3^A: low risk, few symptoms; B: low risk, many symptoms; C: high risk, few symptoms; D: high risk, many symptoms.

SD = standard deviation, IQR = interquartile range.

### Utilities according to GOLD 2013

**Figure 2:**
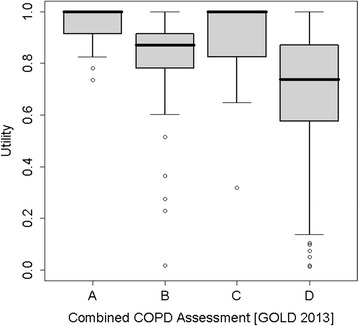
**Utility estimates by GOLD 2013 classification.** LEGEND: **A**: low risk, fewer symptoms; **B**: low risk, more symptoms; **C**: high risk, fewer symptoms, **D**: high risk, more symptoms. Graph shows utilities ≥ 0.

28 (8.1%) patients were classified in group A, 66 (19.1%) in group B, 30 (8.7%) in group C and 222 (64.2%) in group D. Patients with more symptoms presented lower utility values, with patients in group D having the lowest utility. All pairwise comparisons were significant, except for the comparison between groups A and C, maybe as a consequence of a lack of statistical power for this comparison (Table 4 and Figure 2).

GOLD 2013 guidelines recommend the use of CAT or mMRC for assessing symptoms. However, discrepancies were detected in the GOLD 2013 classification depending whether CAT or mMRC scores were used. Therefore, a sensitivity analysis was performed by classifying patients using only CAT or using only mMRC. As in the base case, both analyses showed significant differences in the utility values between groups. Nevertheless, when using only mMRC scale to obtain GOLD 2013 classification, no statistical differences between B and D groups were found (Table 5).

**Table 5:** **Sensitivity analysis of utility estimates by GOLD 2013 classification**

		**Base case**	**Sensitivity analyses**		
		**CAT and mMRC**	**CAT**	**mMRC**	**p-value** ^**3**^
GOLD 2013^2^	***A***				0.058
	N (%)	28 (8.1%)	33 (9.5%)	68 (19.7%)	
	Mean (SD)	0.96 (0.07)	0.94 (0.10)	0.91 (0.09)	
	Median (IQR)	1.00 (0.09)	1.00 (0.09)	0.91 (0.16)	
	***B***				0.04
	N (%)	66 (19.1%)	61 (17.6%)	26 (7.5%)	
	Mean (SD)	0.80 (0.22)	0.80 (0.23)	0.68 (0.30)	
	Median (IQR)	0.87 (0.13)	0.87 (0.13)	0.78 (0.35)	
	***C***				0.2
	N (%)	30 (8.7%)	41 (11.8%)	87 (25.1%)	
	Mean (SD)	0.88 (0.25)	0.86 (0.24)	0.84 (0.22)	
	Median (IQR)	1.00 (0.16)	0.91 (0.18)	0.89 (0.22)	
	***D***				0.1
	N (%)	222 (64.2%)	211 (61.0%)	165 (47.7%)	
	Mean (SD)	0.66 (0.31)	0.65 (0.31)	0.61 (0.32)	
	Median (IQR)	0.74 (0.29)	0.74 (0.31)	0.71 (0.31)	
	p value^1^	<0.001	<0.001	<0.001	

^1^Kruskal-Wallis test and Mann-Whitney U test for post-hoc comparisons (Bonferroni-adjusted).

Base case and sensitivity analysis (CAT): all pairwise comparisons were significant except between A and C; Sensitivity analysis (mMRC): all pairwise comparisons were significant except between A and C, and between B and D.

^2^A: low risk, few symptoms; B: low risk, many symptoms; C: high risk, few symptoms; D: high risk, many symptoms.

^3^Comparison of utilities according to classification by CAT or mMRC. Mann-Whitney U test (without alpha-adjustment).

SD = standard deviation, IQR = interquartile range, CAT = COPD Assessment Test, mMRC = modified Medical Research Council.

### Utilities according to clinical phenotypes

**Figure 3:**
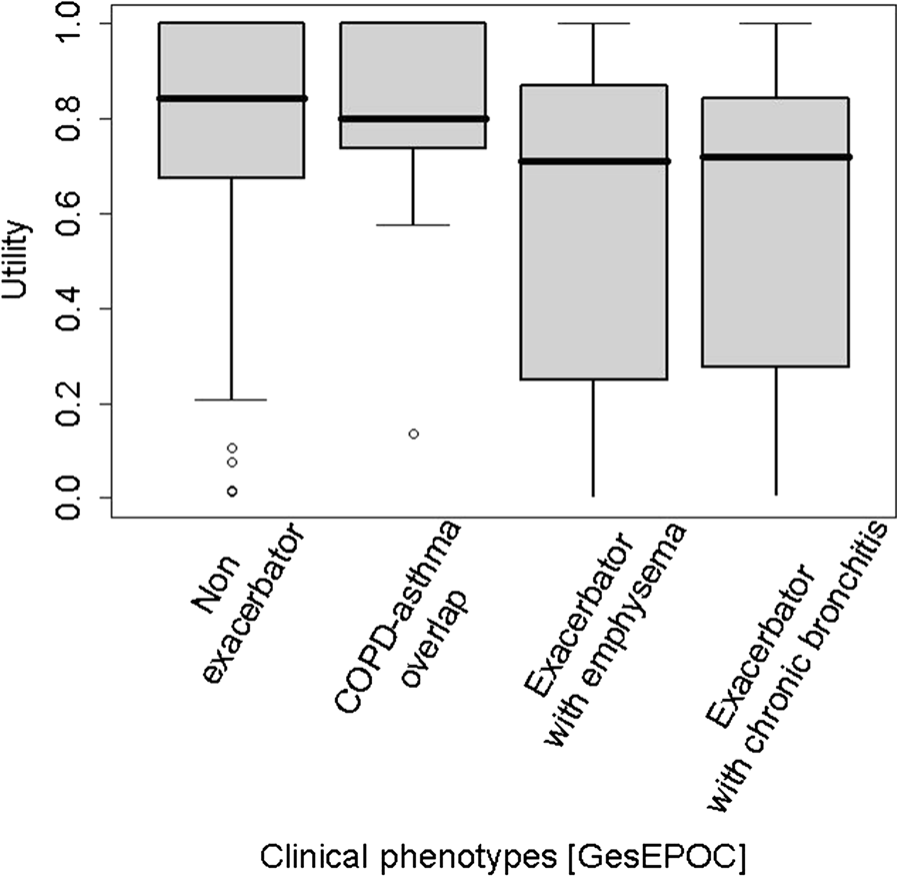
**Utility estimates by GesEPOC clinical phenotypes.** LEGEND: Graph shows utilities ≥ 0.

In 24 cases no phenotype classification was provided by the investigator. Among the 322 patients analysed, 207 (64.3%) were classified as non exacerbator, 21 (6.5%) as COPD-asthma overlap, 41 (12.7%) as exacerbator with emphysema and 53 (16.5%) as exacerbator with chronic bronchitis (Table 4 and Figure 3). The global test showed significant differences (p < 0.001) due to differences between patients with or without exacerbations and between patients with COPD-asthma overlap and those with exacerbations and chronic bronchitis.

### Utilities according to BODEx index

**Figure 4:**
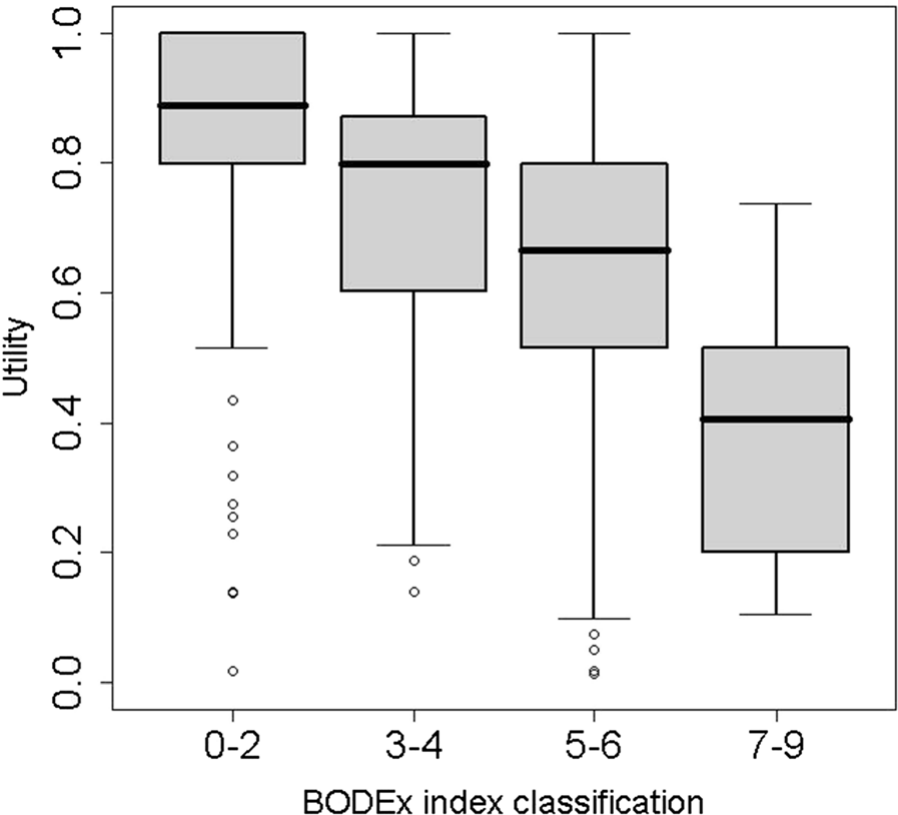
**Utility estimates by BODEx index classification.** LEGEND: Graph shows utilities ≥ 0.

167 (48.3%) patients were classified in the lower severity group (0–2), 93 (26.9%) in the 3–4 interval group, 75 (21.7%) in the 5–6 interval group and 11 (3.2%) in the most severe group (7–9). More severe patients presented lower utility values (p < 0.001). All pairwise comparisons were significant except between the two groups with the greatest severity (Table 4 and Figure 4).

## Discussion

This study estimated generic health utilities in a sample of Spanish COPD patients according to different severity classifications and by clinical phenotype. Irrespective of the classification studied, an association between utilities and disease severity was found, as the health utility of COPD patients decreased as disease severity progressed to more severe states and disease-related symptoms appeared. The primary objective of the study was based on the traditional classification of disease severity, lung function impairment [4]. However, the study also incorporated current recommendations on patient stratification [5,6] being, up to our knowledge, the first study to assess health utilities using these recent classifications.

The results obtained for the primary objective are consistent with those found in other European studies. A review of studies of HRQL measurements using the EQ-5D questionnaire in COPD patients found mean (95% CI) health utilities of 0.74 (0.66, 0.83) in moderate COPD, 0.69 (0.60, 0.78) in severe COPD and 0.61 (0.44, 0.77) in very-severe COPD [12]. The health utilities estimated in the present study were within the confidence intervals presented above.

In Spain, a number of studies have estimated the utility of COPD patients using the EQ-5D questionnaire. The results of the present study were similar to those obtained in a cohort of 115 mild-to-severe COPD patients, 0.72 (SD = 0.31) [29]. The INSEPOC study included 4,574 COPD patients, irrespective of disease severity, with a mean utility of 0.69 (SD = 0.28) [16]. In the cross-sectional observational EPISCAN study [2] the mean utility of 335 COPD patients was 0.86 (0.81, 0.91) somewhat higher than our results, since this was a population-based epidemiological study and most patients were newly diagnosed with mild disease.

Generic questionnaires are suggested to limit discriminatory ability and some studies found little difference in utilities in milder stages of COPD (mild vs moderate and moderate vs severe) [11,30]. In the present study, the sample size was powered to detect differences between the three severity stages and significant differences in utilities were found.

Even though the sample size was calculated to show differences in the primary objective, differences were also found according to GOLD 2013 classification, clinical phenotypes and BODEx index. With respect to GOLD 2013 classification, the three analyses performed (classifying symptoms using CAT and mMRC, only CAT and only mMRC) found statistical differences between patients with fewer or more symptoms (groups A vs B and C vs D). However, in the analysis performed using only mMRC, no significant differences were found between patients at low and high risk (groups A vs C and B vs D), and in the two other analyses differences were found between B and D but not between A and C. These results might suggest that symptoms are better predictors of utilities than lung function or exacerbations. Further studies would be needed to confirm this hypothesis. While the distribution of patients varied in the three GOLD 2013 analyses, the results obtained by evaluating the symptoms using CAT and mMRC were very similar to those obtained using only CAT. However, when only mMRC was considered, the distribution of patients was different to that obtained with the other two analyses, resulting in an estimate of health utilities that also differed. A recent study [31] found a weak association between CAT and mMRC, corroborating these results.

With respect to GesEPOC phenotype classification, significant differences were found between exacerbators and non-exacerbators, corroborating the importance of exacerbations in HRQL. The EPISCAN study estimated the mean utility of COPD-asthma overlap patients (0.76), showing significant differences with non-overlap patients [18]. The present study found not statistically significant differences in mean utility values (0.81 for COPD-asthma overlap, and 0.72 for non-overlap phenotypes, p = 0.07), probably due to a lack of statistical power.

There are no published studies that evaluate the association between BODEx index and utility. However, the analyses performed on the BODE index [32–34] concluded that HRQL deteriorated as the severity of COPD increased. These results are consistent with the results of the present study.

The percentage of patients with the highest-possible utility (value 1) was 22% (moderate COPD 29.6%, severe COPD 20%, very-severe COPD 10.6%), evidencing a ceiling effect, which was also observed in previous studies [12,13,20,27]. One of these studies [13] evaluated EQ-5D questionnaire in 1,235 COPD patients from 13 countries and found that 22.9% patients obtained the highest health utilities (moderate COPD 27.8%, severe COPD 19.7%, very-severe COPD 4.4%). The higher percentage of patients with the highest utility in the very-severe group found in the present study might be due to within-group variability, which is higher than in the other severity groups due to the lower number of very-severe COPD patients.

Generic utilities constitute an essential parameter in health economic evaluations. Most of the published CU analysis in COPD based disease progression on lung function decline, and therefore results of the primary objective could be used to populate future economic analyses. However, due to the multifactorial nature of COPD, utility values estimated in the secondary objectives of this study provide new evidence for the development of new economic models that base disease progression on other clinical variables besides lung function. According to the results of the study, it seems that the GOLD 2007 guidelines and the BODEx index better discriminate patients who have a worse health status than the GOLD 2013 guidelines. At the same time, the GOLD 2013 guidelines are better able to identify a smaller group of patients (GOLD A) with the best health. This may have implications on future economic evaluations of COPD, which could consider different disease severity classifications depending on the main focus of the study. For instance, if the main interest of the economic evaluation is severe or very-severe COPD patients, the GOLD 2007 guidelines might be preferable as they differentiate the health status of these patients better than the GOLD 2013 guidelines. In contrast, the GOLD 2013 guidelines might be preferred in studies whose main focus is on COPD patients with the best health.

The main limitations of the study are related to the sample selection. Firstly, due to the low prevalence of patients with very-severe stable COPD, the number of patients recruited in this category was lower than for the other levels of severity. This may limit the external validity of the results. The sample calculation was, however, sufficient to allow detecting differences between severity levels, and comparison with other studies supports the consistency of the results. On the other hand, due to methodological requirements for utility estimates, patients needed to be stable with no exacerbation in the previous two months. This could have biased the inclusion of GOLD A and B patients and GesEPOC phenotype non exacerbators, and the distribution of patients in the study could differ from the real prevalence. Nevertheless, all groups were sufficiently well-represented in the study to estimate utilities. Finally, 85.5% of the patients enrolled were male. Although this is consistent with the epidemiology of COPD in Spain, the results should be interpreted with caution when extrapolated to females.

## Conclusion

This observational cross-sectional study found an association between generic utilities and all clinical variables used for disease classification (airflow limitation, exacerbation history, symptoms, clinical phenotypes and BODEx index), as the health utility of COPD patients decreased as disease severity progressed to more severe states and disease-related symptoms appeared. GOLD 2007 guidelines and BODEx index better discriminated patients with a worse health status than the GOLD 2013 guidelines, while GOLD 2013 guidelines were better able to identify a smaller group of patients with the best health. Estimated generic utilities for the different COPD stages may be appropriate to model health outcomes in economic evaluations of COPD treatments.
